# Compared Antileishmanial Activity of Clomiphene and Tamoxifen

**DOI:** 10.3390/biomedicines12102290

**Published:** 2024-10-09

**Authors:** Sergio Sifontes-Rodríguez, Alma Reyna Escalona-Montaño, Ricardo Mondragón Flores, Niurka Mollineda-Diogo, Lianet Monzote Fidalgo, Mónica Edith Mondragón-Castelán, Fedra Alardin-Gutiérrez, Lourdes Araceli López-Enzana, Daniel Andrés Sánchez-Almaraz, Ofelia Pérez-Olvera, María Magdalena Aguirre-García

**Affiliations:** 1 Unidad de Investigación UNAM-INC, Facultad de Medicina, Instituto Nacional de Cardiología Ignacio Chávez, Universidad Nacional Autónoma de México, Mexico City 14080, Mexico; almiusqfb23@yahoo.com.mx (A.R.E.-M.); falarding@gmail.com (F.A.-G.); lopezenza.lourdes471@gmail.com (L.A.L.-E.); chillon_15@yahoo.com.mx (D.A.S.-A.); o.perez.olvera@gmail.com (O.P.-O.); 2 Instituto de Investigaciones Biomédicas, UNAM—Consejo Nacional de Humanidades Ciencias y Tecnologías (CONAHCYT), Mexico City 03940, Mexico; 3 Departamento de Bioquímica, Centro de Investigaciones y Estudios Avanzados del IPN, CINVESTAV, Mexico City 07360, Mexico; rmflores@cinvestav.mx (R.M.F.); mmondrag@cinvestav.mx (M.E.M.-C.); 4 Centro de Bioactivos Químicos, Universidad Central “Marta Abreu” de las Villas, Santa Clara 54830, Cuba; niurkam@uclv.cu; 5 Instituto de Medicina Tropical “Pedro Kourí” (IPK), Havana 11400, Cuba; monzote@ipk.sld.cu

**Keywords:** *Leishmania*, clomiphene, tamoxifen, amastigote, promastigote, mouse

## Abstract

Drug repositioning is an efficient strategy to search for new treatment alternatives that is especially valuable for neglected parasitic diseases such as leishmaniasis. Tamoxifen and raloxifene are selective estrogen receptor modulators (SERMs) that have shown antileishmanial activity. Clomiphene is a SERM structurally similar to tamoxifen, whose antileishmanial potential is unknown. That is why the objective of the present work was to evaluate its antileishmanial activity in vitro and in vivo in comparison with tamoxifen. The inhibitory effect against promastigotes of *L. amazonensis*, *L. major,* and *L. mexicana* was evaluated for both compounds, as well as the cytotoxicity against mouse peritoneal macrophages, the growth inhibitory activity in intracellular amastigotes of *L. mexicana*, and the in vivo activity in mice experimentally infected with *L. mexicana*. Clomiphene was about twice as active as tamoxifen against both promastigotes and intracellular amastigotes, with IC_50_ values of 1.7–3.3 µM for clomiphene and 2.9–6.4 µM for tamoxifen against all three species of promastigotes and 2.8 ± 0.2 µM and 3.7 ± 0.3 µM, respectively, against *L. mexicana* amastigotes. Clomiphene structurally affected several parasite organelles in a concentration-dependent fashion, leading to the death of both promastigotes and intracellular amastigotes. Interestingly, the macrophage host cell did not appear damaged by any of the clomiphene concentrations tested. With oral administration at 20 mg/kg for 14 days, both compounds showed similar effects in terms of reducing the growth of the lesions, as well as the weight of the lesions and the parasite load at the end of the follow-up period. The results showed the potential of SERMs as antileishmanial drugs and support further testing of clomiphene and other compounds of this pharmacological group.

## 1. Introduction

Leishmaniasis is a neglected tropical disease caused by parasites of the genus *Leishmania*, transmitted to humans through the bite of infected sandflies. Leishmaniasis manifests in three main forms: cutaneous, mucocutaneous, and visceral (also known as kala-azar). Cutaneous leishmaniasis is characterized by the appearance of skin ulcers and is responsible for 95% of global cases [1]. Mucocutaneous leishmaniasis affects the mucous membranes of the nose, mouth, and throat, while visceral leishmaniasis is the most severe and can be fatal if untreated, affecting internal organs such as the liver and spleen. Every year 50,000–90,000 cases of leishmaniasis occur, mainly in East African countries, Brazil, and India [2].

Leishmaniasis mainly affects poor countries, especially in tropical and subtropical regions. It represents a significant economic, health, and social burden for more than one billion people around the world, especially in low-income countries and the most disadvantaged groups in middle-income countries. The absence of timely access to proper treatment results in many people severely damaged and disfigured, leading to disability and social exclusion [2,3].

The control of leishmaniasis has been complex due to the lack of vaccines, safe and effective drugs, as well as the existence of numerous biological vectors and reservoir hosts with diverse ecological habitats [2]. The efficacy of currently available drugs varies among species of parasites prevalent in different areas, with each species having a unique clinical presentation and resistance profile [4]. Side effects and cost also limit present treatment alternatives [5]. For detailed information on recent advances and trends in vaccine and drug development for leishmaniasis, readers are referred to updated reviews on the topics [6,7,8,9,10,11].

The development of safe and effective antileishmanial vaccines and drugs has been a need for decades. However, the research and development investment needed to bring a new medicine to market has recently been estimated at USD 1335.9 million [12], a figure hardly recoverable with a medication for a disease that mainly affects the poor. In this context, drug repurposing has emerged as an effective way to identify new medicines [7], particularly for leishmaniasis and other neglected tropical diseases [8].

Drug repositioning, also known as drug repurposing or drug re-targeting, involves finding new therapeutic applications for existing drugs. This approach can result in significant savings in the development costs of a drug. The main factors contributing to these savings are (1) Reduction in research and development costs: Since the drug has already gone through the initial discovery and preclinical testing phases, these associated costs and times are saved. (2) Lower risk of failure: Repositioned drugs have already been approved by regulatory agencies for at least one indication, which means their safety and pharmacokinetic profiles are well established. This significantly reduces the risk of failure in the clinical stages. (3) Acceleration of development time: The process of developing new drugs can take 10 to 15 years, while repositioning can reduce this time to 3–5 years, thereby accelerating the availability of the drug for its new indication. (4) Regulatory and clinical trial costs: Although clinical trials are still required to validate the new indication, these are generally less expensive and faster than for a new drug [13,14,15,16].

Selective estrogen receptor modulators (SERMs) are among the many recent attempts of drug repurposing for leishmaniasis. SERMs, primarily known for their use in breast cancer, infertility, and osteoporosis treatments, have been evaluated for their potential activity against *Leishmania* spp. parasites. Tamoxifen and raloxifene are SERMs that have shown antileishmanial activity.

Tamoxifen inhibits growth of promastigotes of several *Leishmania* species in vitro and reduces viability of intracellular amastigotes [17,18]. Its mode of action involves disturbance of sphingolipid metabolism, mitochondrial function, and the cytoplasmic membrane potential [19]. In animal models of cutaneous leishmaniasis, tamoxifen reduces lesion growth and the average number of parasites in the skin lesions [20,21,22]. Moreover, the combination of either amphotericin B and tamoxifen or pentavalent antimonial and tamoxifen has an additive and probably synergistic effect in vivo [23,24]. Furthermore, in mouse and hamster models of visceral leishmaniasis, tamoxifen reduces parasite burden in liver and spleen compared to infected, non-treated animals [25]. 

In cutaneous leishmaniasis patients, co-administration of oral tamoxifen and systemic pentavalent antimonial (meglumine antimoniate) results in higher cure rates in comparison with the standard scheme of treatment [26]. More recently, a combination of a single intramuscular dose of pentamidine followed by oral tamoxifen was compared to three intramuscular doses of pentamidine in a randomized, controlled, open-label, non-inferiority clinical trial. Seventy-two percent of patients allocated to the intervention group and one hundred percent in the control group were cured at six-month follow-up. Although a lower cure rate was achieved with the drug combination, it was safe and considered a promising option for populations in remote areas [27].

Raloxifene has shown antileishmanial effect against promastigotes and intracellular amastigotes of a number of *Leishmania* species, with IC50s of 30.2–38.0 µM and 8.8–16.2 µM, respectively. In BALB/c experimentally infected with *L. amazonensis*, raloxifene reduces lesion size and the number of parasites in lesions [28]. Recent in silico studies suggest that raloxifene could be an inhibitor of trypanothione synthetase, a validated drug target for *Leishmania* spp. [29].

**Figure 1:**
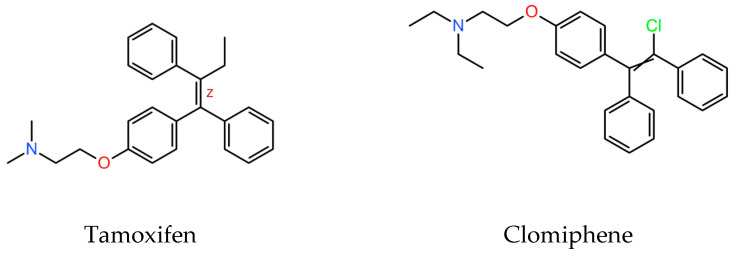
Molecular structure of tamoxifen and clomiphene (downloaded from PubChemCompounds, https://pubchem.ncbi.nlm.nih.gov/, accessed on 15 July 2024).

SERMs are a family of structurally dissimilar compounds at different stages of pharmaceutical development. Among them, clomiphene, (Figure 1) is a triphenylethylene derivative structurally similar to tamoxifen.

Clomiphene is a medication primarily used in the treatment of infertility. In women, it is indicated to induce ovulation, and in men, to induce spermatogenesis, as well as for the treatment of hypogonadism as a means to stimulate endogenous testosterone production. Clomiphene is a safe oral drug that, due to its structural resemblance to tamoxifen, could be a candidate for repositioning for the treatment of leishmaniasis. As far as we know, clomiphene has not been previously tested against *Leishmania* spp. Thus, in the present work, the antileishmanial effect of clomiphene in vitro and in a mouse model of experimental cutaneous leishmaniasis was tested.

## 2. Materials and Methods

### 2.1. Test Compounds 

Tamoxifen and clomiphene (in the form of citrates) were donated by BioCubaFarma (Havana, Cuba) with a purity over 99%. They were dissolved in dimethyl sulfoxide (DMSO, SIGMA-ALDRICH, St. Louis, MO, USA) and conserved at 5 °C until further use. Then, they were dissolved in the different culture media, depending on the assay. The maximum concentration of DMSO in the cultures was 0.5%. Amphotericin B deoxycholate (Gibco, Grand Island, NY, USA), supplied as a 250 µg/mL solution, was used as a positive control.

### 2.2. Parasites

*L. major* MHOM/IL/81/Friedlin and *L. mexicana* MNYC/BZ/62/M379 were donated by Paul A. Bates, Division of Biomedical and Life Sciences, Faculty of Health and Medicine, Lancaster University, UK. *L. amazonensis* MHOM/BR/77/LTB0016 was donated by the Department of Immunology of Fundação Oswaldo Cruz (Fiocruz), Brazil. Cultures of promastigotes were maintained in medium 199 (Gibco, Grand Island, NY, USA) supplemented with 10% fetal bovine serum (Gibco, Grand Island, NY, USA), 1% vitamins (Gibco, Grand Island, NY, USA), and antibiotics (200 IU/mL penicillin and 200 µg/mL streptomycin). Intracellular amastigote cultures were obtained by infection of primary cultures of mouse peritoneal macrophages with axenic *L. mexicana* amastigotes at a ratio of three parasites per macrophage as reported elsewhere [30]. The cultures were maintained at 37 °C and 5% CO_2_ in RPMI-1640 medium (Gibco, Paisley, Scotland, UK).

### 2.3. Animals

Female BALB/c mice, 6–8 weeks old and 16–18 g, were supplied by the Animal Models Unit of the Faculty of Medicine, National Autonomous University of Mexico. They were maintained at 22–25 °C, 60–65% relative humidity, and in a 12 h light/12 h dark light cycle. Mice were handled by trained personnel, and they were sacrificed by CO_2_ inhalation at the end of the assay. The research project was approved by the Committee on the Care and Use of Laboratory Animals (004-CIC-2019). All practices were conducted according to the Mexican Regulation [31] and the Institute of Laboratory Animal Research Guide for the Care and Use of Laboratory Animals [32].

### 2.4. Activity against Promastigotes

The growth inhibition assay was performed according to the procedure of Bodley et al. [33]. Briefly, the promastigotes were cultured in 96-well plates and challenged with serial dilutions of the test (clomiphene and tamoxifen) and control (amphotericin B) compounds. After 48 h at 26 °C, 20 μL of p-nitrophenyl phosphate (20 mg/mL, Sigma-Aldrich, St. Louis, MO, USA) was added to each well and after 3 h at 37 °C the absorbance was read at 405 nm. From the absorbance values and the corresponding concentrations, the mean inhibitory concentrations (IC_50_) were estimated by non-linear fitting to the sigmoid Emax equation [29].

### 2.5. In Vitro Cytotoxicity Assays

Cytotoxicity studies were conducted as described elsewhere [34]. Briefly, primary cultures of mouse peritoneal macrophages were treated with a series of concentrations of the test compounds and were incubated for 48 h at 37 °C and 5% CO_2_. Then, 30 µL of Alamar Blue^TM^ (Thermo Fisher Scientific, Orlando, FL, USA) was added per well. After 6–8 h incubation at 37 °C and 5% CO_2_, fluorescence was read (Ex 485 nm/Em 590 nm) by using a Fluoroskan Ascent FL plate reader (Thermo Labsystems, Waltham, MA, USA). The 50% cytotoxic concentrations (CC_50_) were then estimated from the fluorescence values as indicated above.

### 2.6. Intracellular Amastigote Assay

The antileishmanial activity against intracellular amastigotes was conducted as previously reported [30]. Briefly, cultures of mouse peritoneal macrophages were infected with axenic amastigotes of *L. mexicana*. After 24 h incubation at 37 °C and 5% CO_2_, the culture medium was eliminated and restored with medium containing the test compounds. After another 48 h incubation under the same conditions, the medium was discarded again but replaced with medium 199, and incubation continued for 72 h at 26 °C to allow the surviving amastigotes to transform into promastigotes and proliferate [30]. The number of promastigotes was then determined by addition of p-nitrophenyl phosphate as described above (Section 2.4), and the IC_50_ values were then estimated by non-linear fitting of absorbance versus drug concentration curves to the sigmoid Emax equation [29].

### 2.7. Electron Microscopy Studies

Promastigotes and intracellular amastigotes of *L. mexicana* were exposed for 48 h to concentrations of clomiphene from 0.75 µM to 6 µM and then processed for electron microscopy studies. The final concentration of DMSO in the culture media was 0.1%.

#### 2.7.1. Sample Processing for Scanning Electron Microscopy

Promastigotes were exposed to 1.5 µM to 6 µM clomiphene. Afterwards, they were washed with phosphate-buffered saline (PBS) to remove serum from the culture medium and were subsequently fixed in suspension with 2.5% glutaraldehyde in PBS for 1 h at room temperature (RT). The parasite suspension was washed with PBS at RT and subsequently fixed with 1% osmium tetroxide in PBS for 1 h at 4 °C. The promastigotes were washed exhaustively with PBS at RT. Subsequently, the parasites were adhered to coverslips previously covered with poly-L-lysine (1 mg/mL). The attached parasites were dehydrated by immersion in increasing concentrations of alcohol. They were subsequently critically dried in Samdry-780 A equipment (Tousimis Research, Rockville, MD, USA) and evaporated with gold in a Denton Vacuum Desk II evaporator (Moorestown, NJ, USA). The samples were micrographed on an SEM JSM-6510-LV (JEOL DE MEXICO S.A. DE C.V, Mexico City, Mexico).

#### 2.7.2. Sample Processing for Transmission Electron Microscopy

The promastigotes were fixed with glutaraldehyde and osmium tetroxide as indicated above. Subsequently, they were subjected to a gradual ethanolic dehydration until reaching 100% ethanol. Afterwards, the parasite suspension was gradually infiltrated with Spurr’s resin (EMS, Hatfield, PA, USA). The parasites were polymerized in plastic molds at 60 °C for 72 h. The blocks were cut on an ultramicrotome (Reichert Jung, Vienna, Austria). Thin sections were stained with uranyl acetate and lead citrate and micrographed on a JEM 1400 transmission electron microscope (JEOL DE MEXICO S.A. DE C.V, Mexico City, Mexico).

Intracellular amastigotes were also processed for thin sectioning and transmission electron microscopy. Briefly, macrophages infected and treated wi 0.75 µM to 6 µM clomiphene were washed with PBS, subsequently fixed in 2.5% glutaraldehyde in PBS for 30 min, and then cells were scraped off from the Petri dish and transferred to Eppendorf tubes. The cell pellet was formed by centrifugation at 1500 rpm for 5 min and the fixation time was continued until completing 1 h. The cell pellet was post-fixed in 1% OsO_4_ and dehydrated with increasing concentrations of ethanol and embedded in Spurr´s resin as described above for promastigotes. The ultramicrotome thin sections were stained with uranyl acetate and lead citrate and micrographed under a transmission electron microscope.

### 2.8. In Vivo Antileishmanial Assay

Female BALB/c mice (8/group) were infected in the foot pads with 10^7^ stationary promastigotes of *L. mexicana* per mouse. In this model, lesions develop as a progressive local enlargement of the infected footpad that in chronic stages ulcerates on the plantar aspect and can lead to amputation of the limb. However, within a period of one month after the lesions are evident (as in this study), they usually only develop as a nodule. The experimental protocol conceived that any mouse with lesions with a dorsoplantar diameter over 4.0 mm had to be euthanized.

Once the lesions developed (21 days after inoculation), mice were randomly allocated to experimental groups, and oral treatment with tamoxifen [22] or clomiphene (20 mg/kg, every 24 h, dissolved in isotonic saline solution) was started. One group was treated with amphotericin B as reported elsewhere (7.5 mg/kg, every other day, 7 doses) [35], and one group did not receive any treatment (control group). The lesions were measured weekly for 4 weeks by using a Vernier caliper (Kroeplin, Längenmesstechnick, error 0.05 mm); then, mice were sacrificed, the lesions were excised and weighted, and the parasite load in the lesions of five mice per group were determined with the limiting dilution assay [36].

### 2.9. Statistical Analysis

Results of the in vitro studies were expressed as means ± standard deviations but were not statistically compared due to the reduced number of assays (*n* = 3). The evolution of lesion size over time was analyzed by repeated measure analysis of variance (ANOVA) and Fisher´s Least Significant Difference (LSD) test. Lesion weights were compared by ANOVA and Dunnet´s test (versus non-treated control group). Parasite loads were compared by the Kruskal–Wallis test and the distribution-free multiple comparisons test post hoc. Values of p under 0.05 were considered statistically significant. All analyses were conducted by using GraphPad Prism software (Version 8.0.2, https://www.graphpad.com/).

## 3. Results

In vitro testing of tamoxifen and clomiphene against promastigotes evidenced that clomiphene was near twice more active than tamoxifen for the three tested Leishmania species (Table 1): The IC_50_ values were in the range of 1.7 µM to 3.3 µM for clomiphene, while for tamoxifen they were from 2.9 µM to 6.4 µM. Cytotoxicity for mouse peritoneal macrophages and in vitro activity against intracellular L. mexicana amastigotes were comparable for both compounds. However, the selectivity index of clomiphene was slightly higher than the one of tamoxifen. The activity of amphotericin B against promastigotes and intracellular amastigotes of the three Leishmania species tested was in the range of previously reported IC_50_ values [37,38]. An exact estimate of amphotericin B CC_50_ could not be obtained because it was over the maximum concentration tested, which was achieved with the presentation of amphotericin B used (250 µg/mL) after proper dilution in the culture medium. Nevertheless, the estimate (over 6.7 µM) agreed with Kaiser´s previous report of 23.1 µM [38].

**Figure 2:**
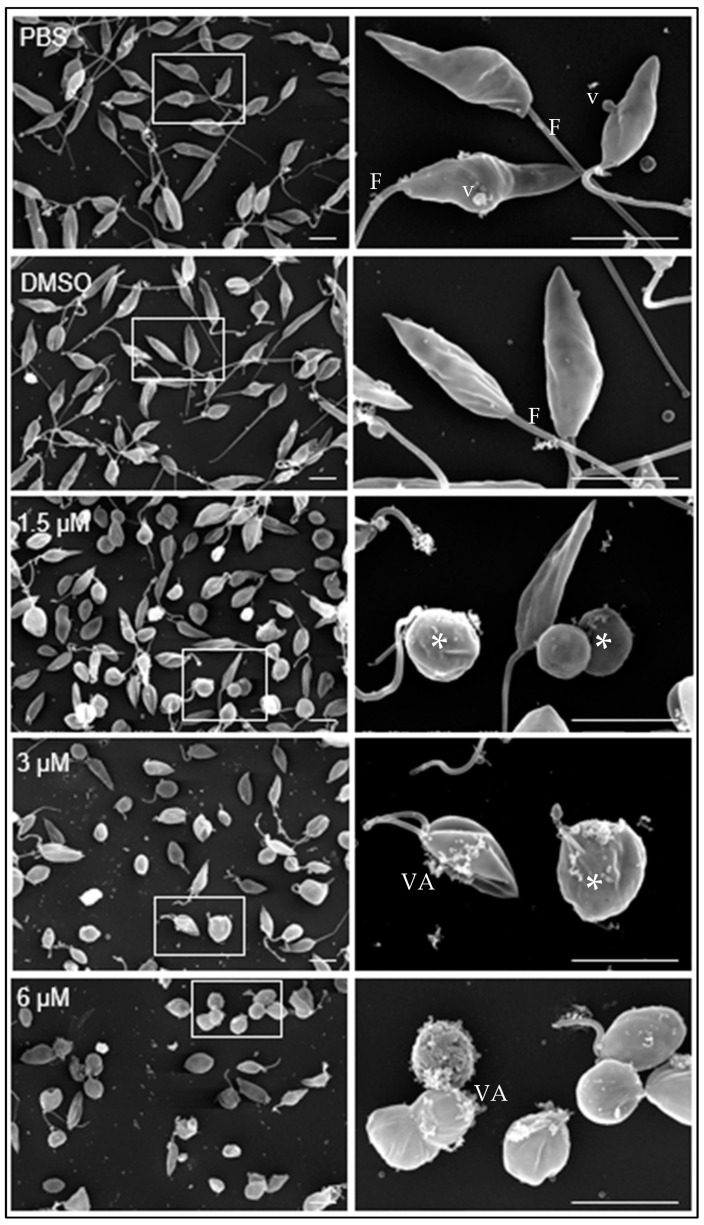
Effect of clomiphene on *L. mexicana* promastigotes treated with clomiphene. Images of scanning electron microscopy on the left side were obtained at low magnification, while on the right side are magnifications of the areas delimited by rectangular frames. *, deformed parasites; F, flagellum; VA, vesicular aggregates; v, vesicles, Scale bar= 500 nm.

Under scanning transmission electron microscopy, promastigotes cultivated in medium containing either PBS or DMSO diluted in PBS showed the typical elongated fusiform shape with one flagellum and cytoplasmic vesicles (v) associated with the plasma membrane (Figure 2, PBS, DMSO).

At 1.5 µM clomiphene (Figure 2, 1.5 µM), two populations of parasites were detected, one that maintained their apparently normal cell shape with elongated flagella and the other population of parasites in evident deformation, with an ovoid or spherical shape that still retained flagella but with atypical folds. At 3 µM clomiphene, most parasites were misshapen and had a spheroidal appearance. All parasites lacked flagella or had very short flagella. Parasites that still retained their elongated shape had a short flagellum attached, no more than 500 nm in length. Additionally, numerous vesicular aggregates, associated with a cottony material (Figure 2, 3 µM), were observed in association with the plasma membrane.

Most promastigotes exposed to 6 µM clomiphene (Figure 2, 6 µM) were deformed and lacked flagella, while only a few showed a short flagellum. The presence of vesicular aggregates and cottony material on the external membrane of the parasites was abundant. Eventually, destroyed parasites were found in the cultures.

**Figure 3:**
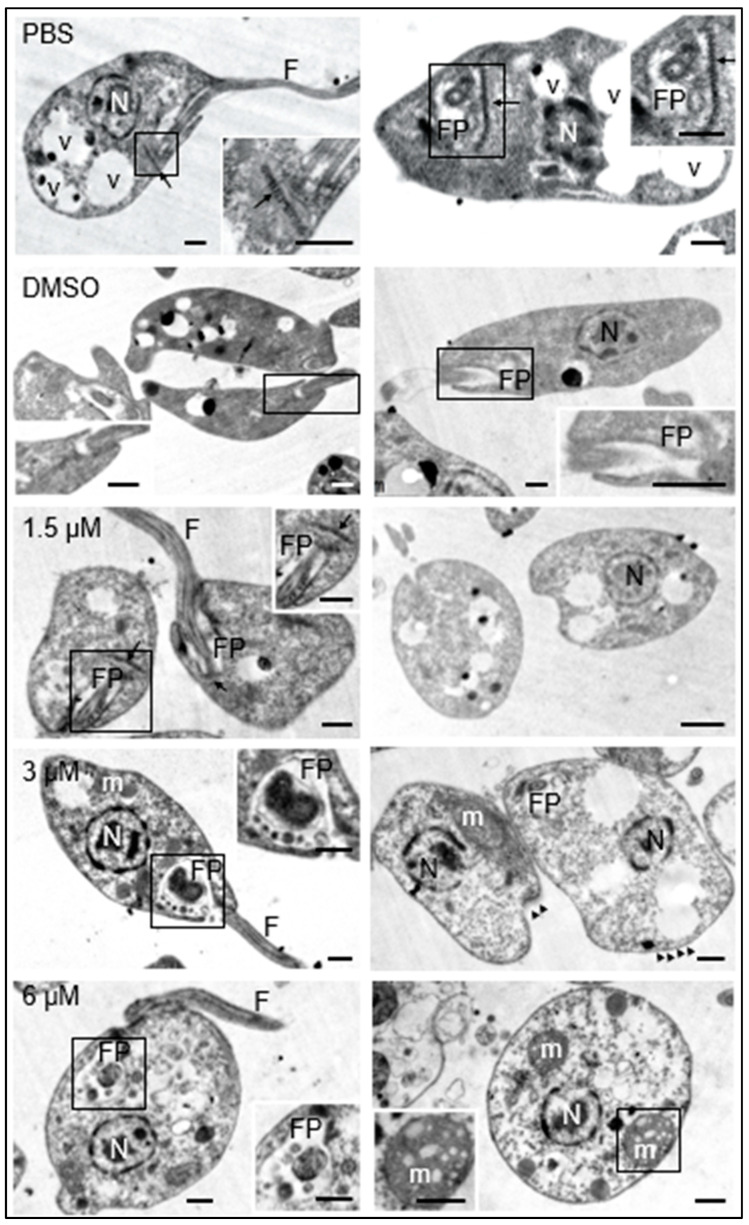
Effects of clomiphene on *L. mexicana* promastigotes visualized by transmission electron microscopy. The micrographs in the left column correspond to low magnification images to show the general structure of promastigotes. The flagellar pockets were framed and magnified to show the changes in these structures. In the right insert of the figure for 6 µM, the structure of a mitochondrion is shown, as well as its respective amplification (insert at bottom left). N, nucleus; FP, flagellar pocket; F, flagellum; m, mitochondrion; v, vesicles; kinetoplast (arrow), microtubules (arrowheads). Scale bar = 500 nm.

Additionally, the morphology of the promastigotes was analyzed under transmission electron microscopy. The promastigotes maintained in culture medium containing 0.1% DMSO did not show structural changes with respect to those maintained in culture medium with the equivalent volume of PBS. These results guaranteed that any changes observed in parasites treated with the test compound were due to its effect and not to DMSO used as a cosolvent. The presence of the nucleus, flagellum, flagellar pocket, kinetoplast, dense granules, and cytoplasmic vesicles was easily distinguishable in both cases (Figure 3, PBS, DMSO).

The exposure of promastigotes to different concentrations of clomiphene resulted in gradual changes in the morphology of various parasite structures as the compound concentration increased. At 1.5 µM clomiphene, swelling of the body of the promastigote began to be detected. Although some parasites presented flagella inserted in the flagellar pockets with presence of apparent normal kinetoplasts, many of them showed ovoid shapes with absence of flagella (Figure 3, 1.5 µM). When promastigotes were treated with 3 µM clomiphene, deformed swollen flagellar pockets containing swollen dense granules of different sizes were detected (Figure 3, 3 µM). In this condition, kinetoplasts were not observed. The flagellum was partially preserved and with an altered association with the flagellar pocket. The mitochondrion was severely deformed and swollen. The cytoplasm showed dense aggregates and some extrusion of its contents. However, the nucleus did not show any apparent morphological alterations.

At the concentration of 6 µM, all the promastigotes were evidently swollen with loss of their typical elongated shape (Figure 3, 3 µM). It was still possible to observe the flagellar pocket, although its content was extruded and showed dense bodies of various sizes. Although it was not possible to visualize the part of the flagellum that is inserted into the flagellar pocket, flagella laterally associated with the deformed parasites were observed. The mitochondrion was evidently swollen and highly dense, with disorganized and swollen mitochondrial cristae. The plasma membrane was apparently intact; however, some parasites were found in an evident process of cell lysis. Dense cytoplasmic granules with abundant vesicles of different sizes were also observed.

**Figure 4:**
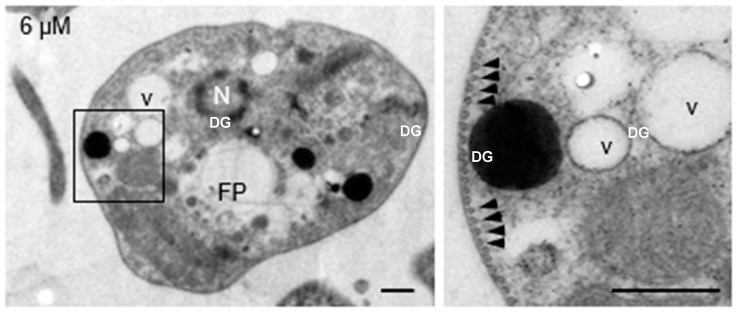
Clomiphene did not affect the integrity of submembrane microtubules. Intact submembrane microtubules (arrowheads) were observed in promastigotes exposed to 6 µM clomiphene. N, nucleus; FP, flagellar pocket; DG, dense granule; v, vesicle. Scale bar = 500 nm.

Submembrane microtubules were not affected by clomiphene treatment even at the highest concentration tested (Figure 4).

In order to determine whether clomiphene had the capacity to affect intracellular parasites located inside the parasitophorous vacuole without damaging the host cell, primary cultures of mouse peritoneal macrophages were infected with *L. mexicana* amastigotes and subsequently exposed to 0.75–6.0 µM clomiphene. As controls, infected macrophages were cultured in medium containing either PBS or 0.1% DMSO (diluted in PBS), as previously explained for promastigotes.

**Figure 5:**
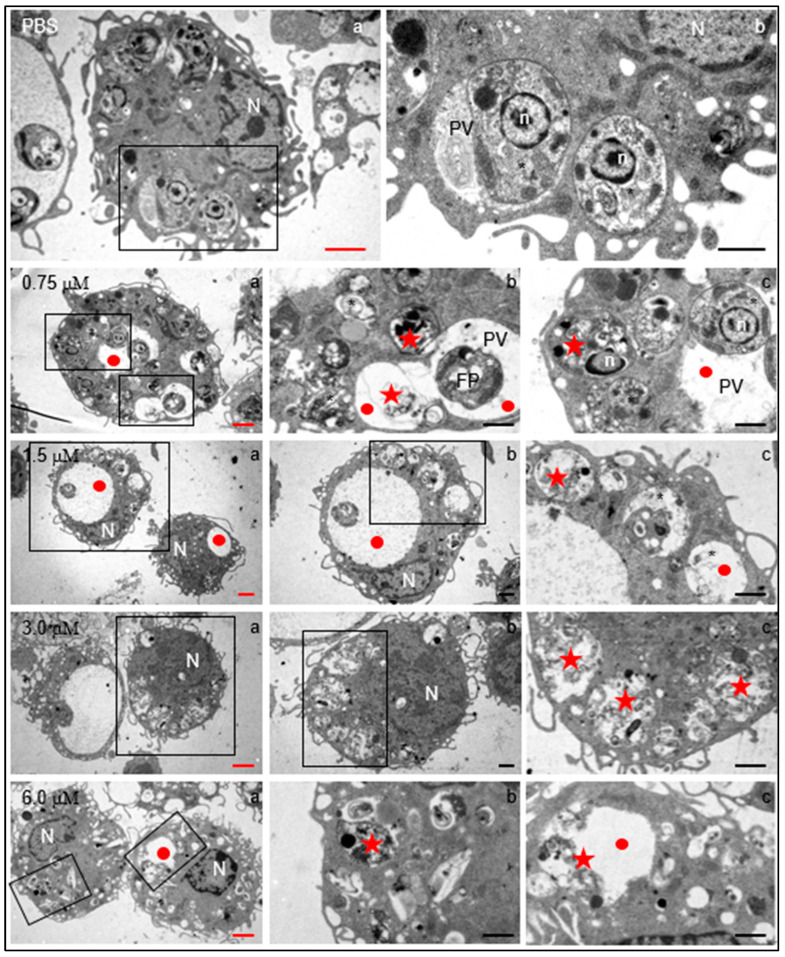
Structural changes induced by clomiphene on intracellular *L. mexicana* amastigotes. Electron transmission microscopy micrographs with red scale bars (subfigures denoted with “a”) correspond to low-magnification images to show the general structure of infected macrophages. Micrographs indicated by black scale bars (subfigures “b” and “c”) correspond to magnifications of framed zones. N, macrophage nucleus; n, amastigote nucleus; PV: parasitophorous vacuole; FP, flagellar pocket; star, intracellular parasites within parasitophorous vacuoles; red circles, enlarged parasitophorous vacuoles; red stars, destroyed parasites within the parasitophorous vacuole. Black scale bars = 2 µm; red scale bars= 1 µm.

Regardless of clomiphene concentration, infected macrophages were apparently normal in terms of nuclear morphology, presence of mitochondria, endoplasmic reticulum, and several filopoidal-type prolongations of the plasma membrane (Figure 5). Moreover, in control cultures, the parasites were found inside the parasitophorous vacuole, showing intact subcellular parasite structures such as the nucleus, mitochondrion, and dense granules. Also, vesicular structures were identified in the intravacuolar space.

Several evident changes were found in infected macrophages exposed to 0.75 µM clomiphene, changes that increased in severity as the concentration of clomiphene was augmented (Figure 5). Among the most evident alterations, an enlargement of the parasitophorous vacuoles which contained membranous and vesicular components was found. Additionally, most of the intravacuolar parasites showed evident structural damage from 0.75 µM concentration. At 3 µM and 6 µM clomiphene, intact parasites were no longer observed and only parasitophorous vacuoles with parasitic detritus were found. Additionally, macrophages showed abundant membrane shunts and cytoplasmic vesicles. Although the macrophages contained destroyed intracellular parasites, the cells were not affected in their integrity nor were they under a process of necrosis or cell death.

In summary, the exposure of parasites to clomiphene caused evident morphological changes, as well as structural alterations of the flagellar pocket, mitochondrion, flagellar length, and loss of cytoplasm. Interestingly, the submembrane microtubules were not affected in their arrangement and integrity. In this sense, clomiphene did not seem to affect the parasite cytoskeleton, but it did affect the organelles involved in its metabolism, thus compromising viability. Clomiphene, at all the tested concentrations, destroyed intracellular amastigotes residing within the parasitophorous vacuole. On the contrary, macrophages were apparently not affected by the compound in their viability or cell integrity, a fact that supports the selective antileishmanial effect of clomiphene.

### In Vivo Antileishmanial Assay

**Figure 6:**
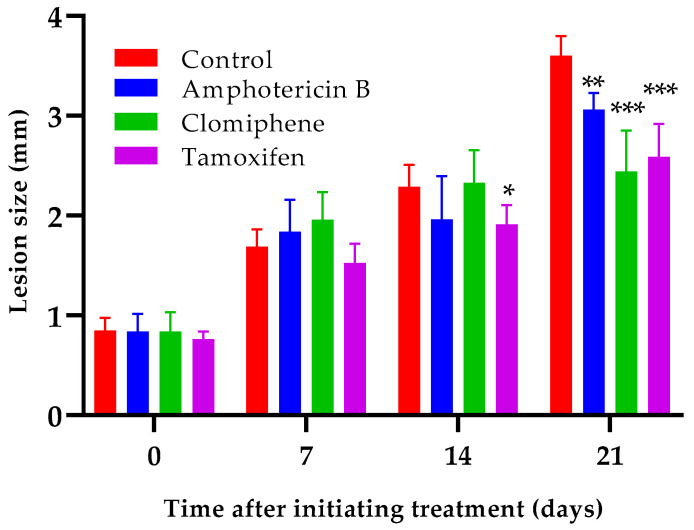
Effect of clomiphene and tamoxifen treatment on lesion growth. *: *p* < 0.05, **: *p* < 0.01, ***: *p* < 0.001 (compared to the control group).

Oral administration of either clomiphene or tamoxifen at 20 mg/kg by oral route reduced lesion growth compared to non-treated infected control mice (Figure 6). Statistically significant differences (*p* < 0.001) between the size of the lesions of any of the treated groups and those of the control mice were evident one week after completing treatment (day 21 after the start of treatment). Interestingly, the lesion sizes of mice treated with either clomiphene or tamoxifen were smaller (*p* = 0.037 for clomiphene and *p* = 0.050 for tamoxifen) than those of amphotericin B-treated mice at day 21, although amphotericin B was much more active in vitro than clomiphene and tamoxifen.

We have previously demonstrated that amphotericin B at doses of 7.5–12.5 mg/kg in mice experimentally infected with *L. amazonensis* not only retards lesion growth but also reduces lesion size [35]. Therefore, higher amphotericin B activity was expected with the dose of 7.5 mg/kg used in the present experiment. Considering that the strains of L. mexicana and L. amazonensis used were similarly susceptible to amphotericin B (Table 1), we speculate that the lower in vivo activity observed here could be due to a higher virulence of L. mexicana for BALB/c mice compared to that of L. amazonensis.

**Figure 7:**
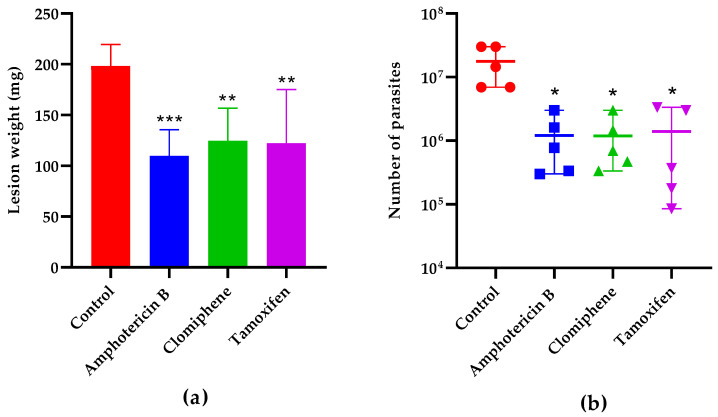
Effect of treatment on lesion weight (**a**) and parasite load (**b**) one week after the end of treatment. *: *p* < 0.05, **: *p* < 0.01, ***: *p* < 0.001 (compared to the control group).

The lesion weight one week after the end of treatment (Figure 7a) was also statistically smaller in mice treated with either clomiphene (*p* = 0.0043), tamoxifen (*p* = 0.0031), or the reference drug amphotericin B (*p* = 0.0007) compared to non-treated controls. The number of parasites in the lesions was also reduced in treated mice compared to controls (Figure 7b). Notably, the lesion weight and the parasite load were comparable (*p* > 0.05) in mice treated with either clomiphene, tamoxifen, or amphotericin B.

## 4. Discussion

SERMs, and triphenylethylene derivatives in particular, are considered a privileged family of compounds, since they have shown activity against bacteria, fungi, viruses, and parasites, besides their primary indications as estrogen modulators [39]. Despite the variety of SERMs partially or fully developed as drugs, only two of them, tamoxifen and raloxifene, have been tested for their potential as antileishmanial agents.

In the present work, another SERM, clomiphene, demonstrated leishmanicidal activity against promastigotes of three *Leishmania* species and against intracellular *L. mexicana* amastigotes, with IC_50_ values slightly lower than those of tamoxifen tested in parallel. The selectivity index of clomiphene was also slightly better than that of tamoxifen and the activity in a mouse model of experimental cutaneous leishmaniasis by *L. mexicana* was similar for both compounds.

Electron microscopy studies confirmed the selective leishmanicidal activity of clomiphene, since morphological changes of the amastigotes were observed at concentrations as low as 0.75 µM, but macrophages, the host cells, remained unaltered at the highest tested concentration (6.0 µM). Both tamoxifen and raloxifene alter mitochondrial function and morphology and eventually lead to cell death without primarily affecting cell membrane permeability and integrity [24,40]. Similarly, in cultures exposed to clomiphene, amastigotes with severely affected organelles (including mitochondrion) with apparently intact cytoplasm membranes were observed.

Regarding the antileishmanial mechanism of action of tamoxifen, it induces alkalinization of the phagolysosome [20]. The acidic pH in normally functioning phagolysosomes is the proper environment for the intracellular transformation of *Leishmania* promastigotes into amastigotes and for their proliferation and survival; therefore, the tamoxifen-induced alkalinization of the phagolysosome reduces amastigote multiplication and viability. Tamoxifen also interferes in sphingolipid biosynthesis of *Leishmania* [41].

Sphingolipids control several cell processes and are components of *Leishmania* cell membranes [42,43,44]. Inositol phosphorylceramide is the main sphingolipid in *Leishmania* but is absent in mammalian cells, resulting in an efficient antileishmanial drug target [44,45]. Moreover, tamoxifen causes mitochondrial disruption and loss of membrane potential. It also produces cell membrane depolarization without affecting its integrity and selective permeability [40].

On the other hand, raloxifene depolarizes mitochondrial and plasma membrane potentials of *Leishmania* spp., resulting in functional disorders on the plasma membrane and the mitochondrion, which culminate in cell death. Notably, treated parasites display autophagosomes and mitochondrial damage, while the plasma membrane remains continuous [28]. Considering the similarity of the ultrastructural effects induced by tamoxifen and raloxifene with those caused by clomiphene, the mechanism of action of clomiphene probably shares at least some characteristics with that of these two other compounds.

Combination therapy is a potentially efficient approach to identify new alternatives for the treatment of leishmaniasis. Combining two or more drugs with either additive or, ideally, synergistic antileishmanial effect reduces the probability of the parasite to develop resistance, as well as the doses required, costs, and toxicity. It could also shorten treatment duration and improve patient compliance.

The combination of tamoxifen with other antileishmanial drugs has been tested experimentally and clinically. The combination with meglumine antimoniate [23] was more promising than that of tamoxifen + amphotericin B [24] or tamoxifen + miltefosine [46]. In a pilot clinical trial, oral tamoxifen in combination with meglumine antimoniate (58% efficacy) was not statistically superior (*p* = 0.82) to meglumine antimoniate alone (40% efficacy), probably due to the reduced number of patients (15 and 12, respectively), but showed favorable results [26]. On the contrary, the combination of pentamidine and tamoxifen had lower clinical efficacy than pentamidine alone (72% vs. 100% efficacy) [27]. Testing the combination of clomiphene with standard antileishmanial drugs (amphotericin B, miltefosine, pentamidine and meglumine antimoniate) is needed.

Tamoxifen and clomiphene, although used in different contexts, have some common side effects due to their action on estrogen receptors. They both can cause hot flashes, nausea, headaches, and cataracts; affect mood; and have effects on the reproductive system (tamoxifen can cause menstrual changes and clomiphene can induce multiple ovulation). However, tamoxifen has serious risks such as thrombosis, strokes, endometrial cancer and hepatotoxicity, while clomiphene can cause ovarian hyperstimulation syndrome and multiple pregnancies. Tamoxifen is more associated with long-term adverse effects such as cardiovascular risks and endometrial cancer, while adverse effects of clomiphene are usually more immediate and related to ovulation and fertility. Clomiphene and tamoxifen are generally considered safe medications; however, the safety profile of clomiphene (mainly with respect to serious unwanted effects) is more favorable than that of tamoxifen [47,48].

## 5. Conclusions

Clomiphene showed in vitro and in vivo activity comparable to that of tamoxifen. Likewise, electron microscopy studies demonstrated that clomiphene has a selective leishmanicidal effect, since it causes concentration-dependent structural changes in the parasite without affecting the host cell. Considering that the safety profile of clomiphene is more favorable than that of tamoxifen, repurposing clomiphene as an antileishmanial agent could even be a more attractive option. Results presented in this paper and those of previous studies support future research on the antileishmanial potential of selective estrogen receptor modulators.

## Figures and Tables

**Table 1:** In vitro antileishmanial activity and cytotoxicity of clomiphene and tamoxifen.

Compound	Promastigotes IC_50_ ± SD (µM)			Cytotoxicity CC_50_ ± SD (µM)	Amastigotes *L. mexicana* IC_50_ ± SD (µM)	S.I.
	*L. mexicana*	*L. major*	*L. amazonensis*			
Tamoxifen	6.4 ± 2.1	2.9 ± 1.1	5.3 ± 0.5	18.8 ± 0.2	3.7 ± 0.3	5.1
Clomiphene	3.0 ± 0.6	1.7 ± 0.9	3.3 ± 0.8	19.8 ± 2.8	2.8 ± 0.2	7.1
Amphotericin B	0.039 ± 0.009	0.030 ± 0.002	0.028 ± 0.004	>6.7 (22.4) *	0.29 ± 0.02	>23.1 (77) **

S.I. = CC_50_/IC_50amastigotes_. * CC_50_ value reported by [38]. ** Selectivity index calculated based on CC_50_ reported by [38].

## Data Availability

The raw data supporting the conclusions of this article will be made available by the authors on request.
